# Nomogram predicting long-term overall and cancer-specific survival of patients with buccal mucosa cancer

**DOI:** 10.1186/s12903-022-02147-9

**Published:** 2022-04-22

**Authors:** Fengze Wang, Jiao Wen, Shuaishuai Cao, Xinjie Yang, Zihui Yang, Huan Li, Haifeng Meng, Florian M. Thieringer, Jianhua Wei

**Affiliations:** 1 State Key Laboratory of Military Stomatology and National Clinical Research Center for Oral Diseases and Shaanxi Clinical Research Center for Oral Diseases, Department of Oral and Maxillofacial Surgery, School of Stomatology, The Fourth Military Medical University, Xi’an, China; 2 MIRACLE Smart Implants Group, Department of Biomedical Engineering, University of Basel, Allschwil, Switzerland; 3 Medical Additive Manufacturing Research Group, Department of Biomedical Engineering, University of Basel, Allschwil, Switzerland; 4 State Key Laboratory of Military Stomatology and National Clinical Research Center for Oral Diseases and Shaanxi Engineering Research Center for Dental Materials and Advanced Manufacture, Department of Anesthesiology, School of Stomatology, The Fourth Military Medical University, Xi’an, China; 5 Department of Stomatology, Shenzhen University General Hospital and Shenzhen University Clinical Medical Academy, Shenzhen University, Shenzhen, China; 6 Department of Stomatology, Binzhou People’s Hospital, Binzhou, China; 7 Clinic of Oral and Cranio-Maxillofacial Surgery, University Hospital Basel, Basel, Switzerland

**Keywords:** Buccal mucosa cancer (BMC), Nomogram, Overall survival, Cancer-specific survival, Decision curve

## Abstract

**Background:**

Few models about the personalized prognosis evaluation of buccal mucosa cancer (BMC) patients were reported. We aimed to establish predictive models to forecast the prognosis of BMC patients.

**Methods:**

The complete clinicopathological information of BMC patients from the surveillance, epidemiology and end results program was collected and reviewed retrospectively. Two nomograms were established and validated to predict long-term overall survival (OS) and cancer-specific survival (CSS) of BMC patients based on multivariate Cox regression survival analysis.

**Results:**

1155 patients were included. 693 and 462 patients were distributed into modeling and validation groups with 6:4 split-ratio via a random split-sample method. Based on the survival analysis, independent prognostic risk factors (variables that can be used to estimate disease recovery and relapse chance) influencing OS and CSS were obtained to establish nomograms. Then, we divided the modeling group into high- and low-risk cohorts. The low-risk cohort had improved OS and CSS compared to the high-risk cohort, which was statistically significant after the Log-rank test (*p* < 0.05). Furthermore, we used the concordance index (C-index), calibration curve to validate the nomograms, showing high accuracy. The decision curve analyses (DCA) revealed that the nomograms had evident clinical value.

**Conclusions:**

We constructed two credible nomogram models, which would give the surgeons reference to provide an individualized assessment of BMC patients.

**Supplementary Information:**

The online version contains supplementary material available at 10.1186/s12903-022-02147-9.

## Background

Oral squamous cell carcinoma (OSCC) ranks sixth among all the cancer categories [1]. OSCC mainly includes tongue, buccal, floor of mouth, hard and soft palate cancers [2, 3]. Frequently, BMC accounts for the second or third proportion of OSCC, especially among the population of Southeast Asia due to the habit of chewing betel quid [1]. The buccal mucosa is adjacent to bone, skin and mastication muscles, leading to early involvement of these sites in BMC. This pattern contributes to a large percentage of BMC being categorized as T4 stage [4, 5]. In almost all studies the prognosis of buccal cancer is poor because of its aggressive tumor behavior and high local recurrence rate. However, compared with other oral cancers, few studies comprehensively evaluate the prognosis of patients with BMC [6]. Generally, most researches assess how different BMC characteristics and treatment influence the prognosis of patients with BMC [7]. Numerous researches have reported the prognosis of the general OSCC [8]. However, few studies regarding BMC patients’ prognosis were documented. Thus, more emphasis should be laid on the prognosis research of patients with BMC.

National Comprehensive Cancer Network (NCCN) clinical guideline suggests assessing the prognosis of OSCC patients via the 7th American Joint Committee on Cancer (AJCC) Staging Manual merely based on TNM staging [9]. However, other relevant clinicopathological parameters also influence the prognosis of the BMC patients such as age, tumor size, tumor thickness, neck nodal metastasis, surgical margin, and grade [10, 11]. Hence, taking the relevant factors into account would provide an accurate prediction of the prognosis of BMC patients. The OS and CSS nomogram is a novel tool to predict the personalized prognosis evaluation.

Nomogram can transform the independent risk factors from Cox regression survival analysis into visual graphics [12], which was widely applied to conduct personalized prognosis evaluation including prostate cancer [13], pulmonary adenocarcinoma [14], oral squamous cell carcinoma [15], oropharyngeal squamous cell carcinoma [16]. Most importantly, the 8th version of the AJCC staging manual recommended that the future version would embrace the nomogram to realize the pursuit of personalized medicine [17]. We sought to establish two nomograms forecasting long-term OS and CSS of the BMC patients by integrating diverse prognostic variables obtained from the Kaplan–Meier and Cox regression model.

## Methods

### Patients’ characteristics and survival analysis

The study was approved by the Ethic Committee of Stomatological Hospital of the Fourth Military Medical University (Approval number: IRB-REV-2020059). We collected the detailed clinicopathological information of 1155 patients with buccal cancer, from the years 2004–2013, from the SEER database: http://seer.cancer.gov. The inclusion criteria were as follows: clear tumor location; detailed clinicopathological information and active OS and CSS follow-up data. Data collected from death certificate or autopsy was excluded. 1155 BMC patients’ detailed information was collected from the SEER database including age, sex, race, origin, grade, surgery, radiation, T stage, N stage, M stage, OS, CSS and survival time (Table 1). The original variable included non-spanish-hispanic-latino and spanish-hispanic-latino. The meanings of grade I, II, III, IV were well differentiated, moderately differentiated, poorly differentiated and undifferentiated respectively. Based on SAS variables “sur_time_mon”, “STAT_REC”, “VSRTSADX” in the SEER database, we obtained patients’ OS, CSS and survival time information. The OS duration time was determined as the period from the diagnosis to death or the last follow-up time. However, CSS focused on the death caused by BMC only.

**Table 1:** Patients’ detailed data

Variables	Modeling group (n = 693)		Validation group (n = 462)	
	n	%	n	%
*Age*				
< 35	21	3.0	14	3.0
36–45	51	7.4	37	8.0
46–55	124	17.9	80	17.3
56–65	156	22.5	96	20.8
66–75	162	23.4	107	23.2
76–85	120	17.3	93	20.1
85 +	59	8.5	35	7.6
*Sex*				
Male	379	54.7	251	54.3
Female	314	45.3	211	45.7
*Race*				
White	530	76.5	354	76.6
Black	67	9.7	38	8.2
Others	96	13.9	70	15.2
*Origin*				
NSHL	632	91.2	424	91.8
SHL	61	8.8	38	8.2
*Grade*				
I	211	30.4	140	30.3
II	367	53.0	240	51.9
III	112	16.2	77	16.7
IV	3	0.4	5	1.1
*Surgery*				
Performed	585	84.4	394	85.3
None	108	15.6	68	14.7
*Radiation*				
Yes	335	48.3	225	48.7
No	358	51.7	237	51.3
*T stage*				
T1	272	39.2	170	36.8
T2	231	33.3	160	34.6
T3	87	12.6	48	10.4
T4	103	14.9	84	18.2
*N stage*				
N0	459	66.2	313	67.7
N1	101	14.6	61	13.2
N2	129	18.6	86	18.6
N3	4	0.6	2	0.4
*M stage*				
M0	680	98.1	454	98.3
M1	13	1.9	8	1.7

NSHL: Non-Spanish-Hispanic-Latino. SHL: Spanish-Hispanic-Latino. Grade I: Well differentiated. II: Moderately differentiated. III: Poorly differentiated. IV: Undifferentiated

According to the random split-sample method, we divided the patients into modeling group (n = 693) and validation group (n = 462). Based on the modeling group, we conduct the univariate OS and CSS analysis and log-rank test firstly. The variables with statistical significance were incorporated into the multivariate Cox regression model to determine the final independent prognostic risk indicators secondly via SPSS 21.0 software for windows [18]. Two-side *p* value was applied and *p* < 0.05 was considered statistically significant.

### Nomogram construction and risk classification

We integrated all the independent prognostic risk factors to construct the nomogram through the “rms” package of the R. 3.2.4 software, which can transform the clinicopathological information into linear graphs. In the graph, every indicator axis was assigned a corresponding score according to every single patient’s information. Thus, each patient’s total scores were calculated and the modeling group’s patients were divided into high- and low-risk cohorts according to cut-off value via R survminer and maxstat packages. We compared the OS and CSS of the above two cohorts via Kaplan–Meier and Log-rank tests.

### Nomogram validation

We used 1000 resamples bootstrapping and ten-fold cross-validation method to conduct internal and external validation [12]. C-index and calibration curves were applied to evaluate the accuracy of the nomogram model. The calibration curves included two main lines: the 45-degree reference line and the actual line. The distance between the above two lines reflected the precision of the model. Moreover, decision curves were plotted to mirror the clinical value of the predicted nomogram model. In the decision curve, the abscissa and ordinate represented the threshold probability and net benefit respectively. The horizontal line and oblique line indicated that all samples were negative and positive accordingly, corresponding to a different net benefit.

The C-index, calibration curve and decision curve were realized using “Hmisc”, “rms” and “stdca.R” packages.

## Results

### Patients’ characteristics and survival analysis

693 and 462 patients were assigned into the modeling and validation groups, applying the random split-sample method with 6:4 split-ratio. In the modeling group, 379 were male and 530 patients were white. 61 patients’ origin was Spanish-Hispanic-Latino. In addition, 367 and 211 patients had moderate and well differentiated buccal carcinomas respectively. Of these modeling group, 585 and 335 patients received surgery and radiotherapy respectively. What’s more, patients with T1–T2 stage accounted for 72.5%. The proportion of patients with N1-3 stage and M1 stage was 33.8% and 1.9% respectively. Also, the statistic for the validation group were given in Table 1.

The modeling and validation groups’ median follow-up times were 24 months (0–119 months) and 23 months (0–119 months) respectively. In the modeling group, 277 patients died in the latest follow-up. 202 patients died of BMC. In addition, 75 patients died of other reasons rather than BMC.

Among the modeling group, the Kaplan–Meier univariate survival analysis showed that age, race, grade, surgery, radiation, T stage, N stage and M stage were related elements affecting OS (*p* < 0.05). Cox multivariate regression analysis revealed that age, grade, surgery, T stage and N stage were independent prognostic risk factors (*p* < 0.05). Moreover, about CSS analysis, age, grade, surgery, T stage, N stage and M stage were independent prognostic risk factors after Kaplan–Meier and Cox multivariate regression analysis. The detailed statistical data was shown in Tables 2 and 3.

**Table 2:** OS analysis in modeling group

Variables	Univariate analysis	Multivariate analysis	
	*p* Value	HR(95% CI)	*p* Value
*Age*	< 0.001		< 0.001
< 35		0.110(0.034–0.363)	< 0.001
36–45		0.189(0.099–0.360)	< 0.001
46–55		0.263(0.165–0.418)	< 0.001
56–65		0.307(0.199–0.474)	< 0.001
66–75		0.418(0.280–0.623)	< 0.001
76–85		0.525(0.349–0.791)	0.002
85 +		Reference	
*Race*	< 0.001		0.791
White			
Black			
Others			
*Grade*	< 0.001		< 0.001
I		0.048(0.011–0.212)	< 0.001
II		0.054(0.012–0.236)	< 0.001
III		0.072(0.016–0.317)	0.001
IV		Reference	
*Surgery*	< 0.001		< 0.001
Performed		Reference	
None		2.549(1.842–3.528)	< 0.001
*Radiation*	< 0.001		0.276
Yes			
No			
*T stage*	< 0.001		< 0.001
T1		0.513(0.340–0.773)	0.001
T2		0.651(0.454–0.933)	0.019
T3		0.909(0.595–1.388)	0.658
T4		Reference	
*N stage*	< 0.001		< 0.001
N0		0.135(0.047–0.383)	< 0.001
N1		0.287(0.100–0.821)	0.020
N2		0.392(0.139–1.107)	0.077
N3		Reference	
*M stage*	< 0.001		0.067
M0			
M1			

Others: American Indian/AK Native, Asian/Pacific Islander. Grade I: Well differentiated. II: Moderately differentiated. III: Poorly differentiated. IV: Undifferentiated

**Table 3:** CSS analysis in modeling group

Variables	Univariate analysis	Multivariate analysis	
	*p* Value	HR(95% CI)	*p* Value
*Age*	< 0.001		< 0.001
< 35		0.239(0.070–0.825)	0.023
36–45		0.378(0.187–0.762)	0.007
46–55		0.467(0.266–0.821)	0.008
56–65		0.470(0.269–0.821)	0.008
66–75		0.718(0.430–1.200)	0.206
76–85		0.704(0.411–1.205)	0.201
85 +		Reference	
*Grade*	< 0.001		< 0.001
I		0.040(0.009–0.185)	< 0.001
II		0.044(0.010–0.200)	< 0.001
III		0.058(0.013–0.265)	< 0.001
IV		Reference	
*Surgery*	< 0.001		< 0.001
Performed		Reference	
None		2.560(1.780–3.681)	< 0.001
*Radiation*	< 0.001		0.296
Yes			
No			
*T stage*	< 0.001		0.001
T1		0.421(0.260–0.680)	< 0.001
T2		0.596(0.398–0.894)	0.012
T3		0.929(0.583–1.479)	0.755
T4		Reference	
*N stage*	< 0.001		< 0.001
N0		0.163(0.049–0.541)	< 0.001
N1		0.415(0.125–1.379)	0.151
N2		0.563(0.172–1.845)	0.343
N3		Reference	
*M stage*	< 0.001		0.040
M0		0.490(0.249–0.968)	
M1		Reference	

Grade I: Well differentiated. II: Moderately differentiated. III: Poorly differentiated. IV: Undifferentiated

### Nomogram construction and risk classification

**Fig. 1:**
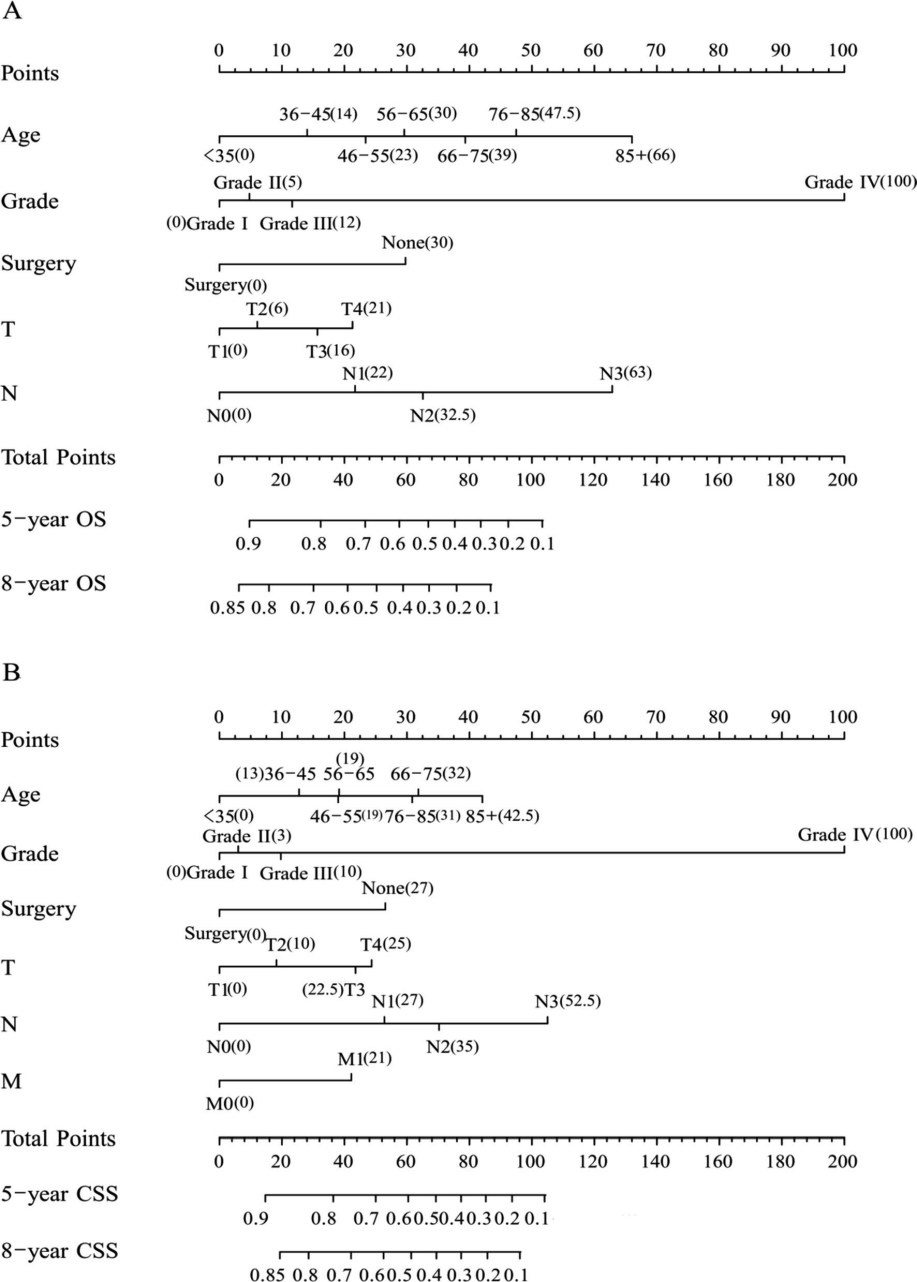
Nomogram forecasting long-term OS (**A**) and CSS (**B**) of patients with BMC. The nomogram scores for each subcategory of the clinical parameters are shown in brackets

**Fig. 2:**
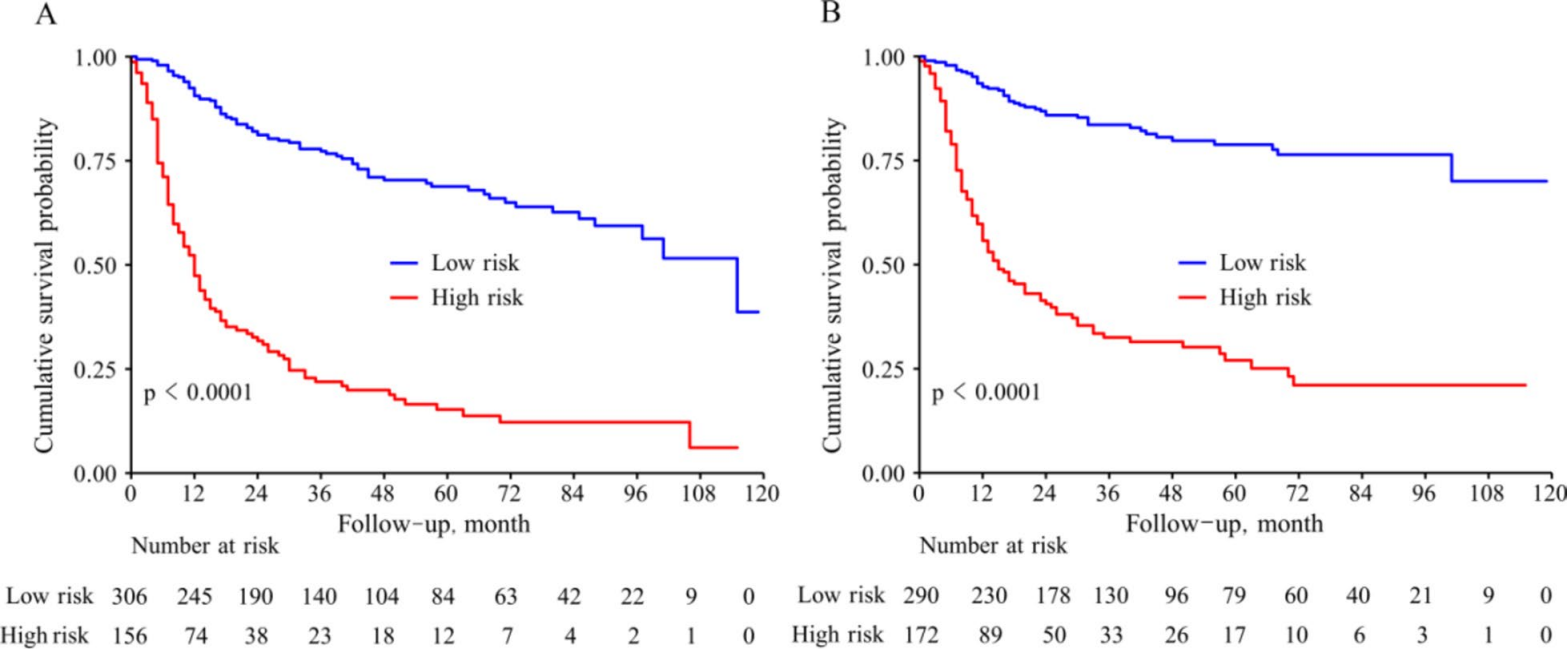
Survival curves for high- and low-risk group patients according to nomogram scores. **A** For OS. **B** For CSS

We incorporated the above OS- and CSS-relevant prognostic risk factors to construct the nomograms, which was shown in Fig. 1. Based on the scores calculated according to nomograms, we obtained the cut-off values to divide the patients into high- and low-risk parts. The cut-off values regarding OS and CSS were 73 and 58 respectively. The survival curves were shown in Fig. 2.

### Nomogram validation

**Fig. 3:**
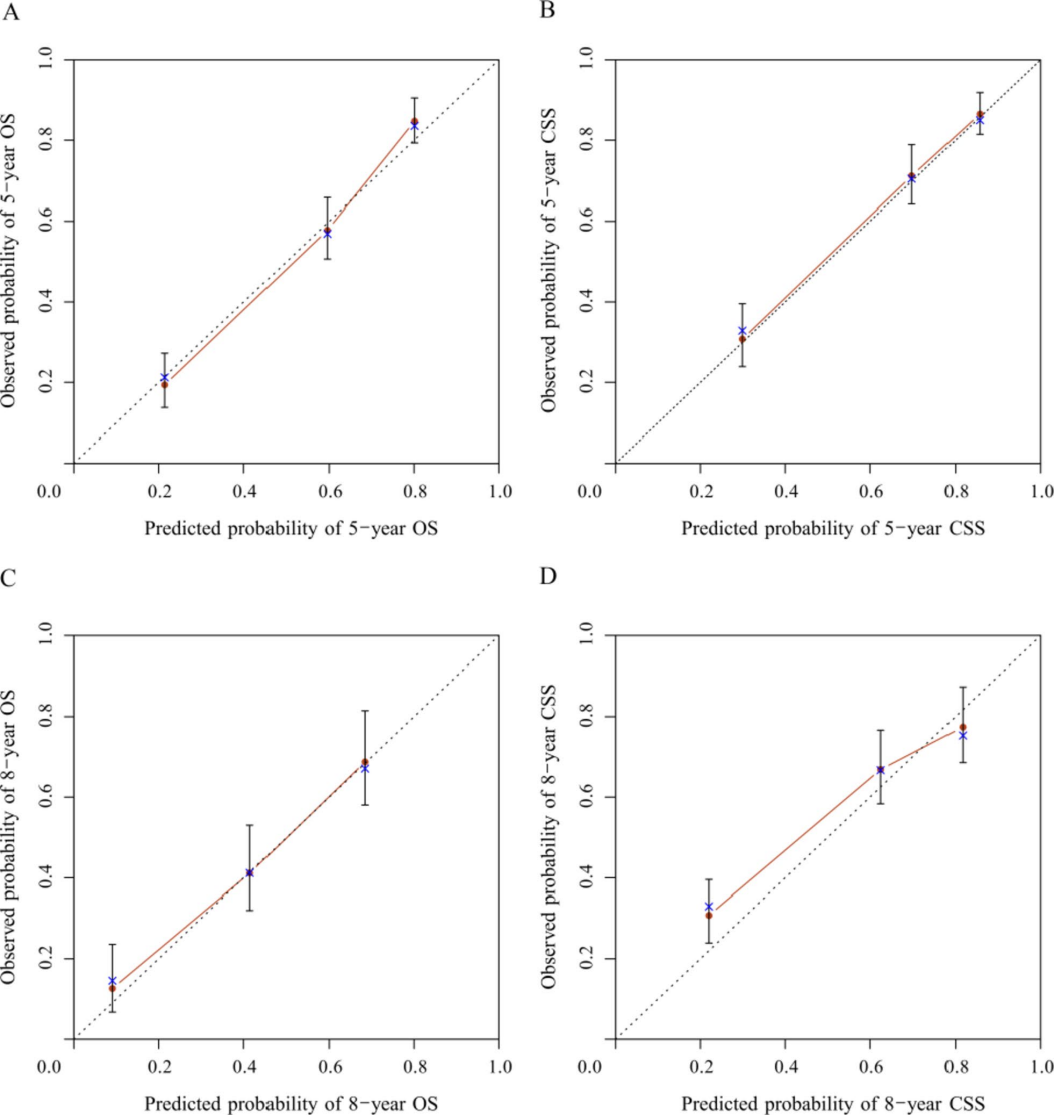
Calibration curves for internal validation for long-term OS (**A**, **C**) and CSS (**B**, **D**)

**Fig. 4:**
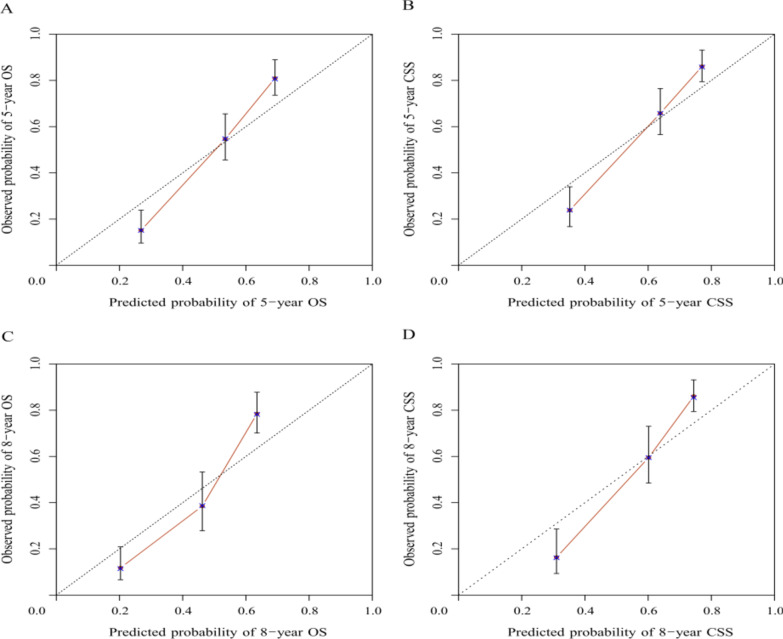
Calibration curves for external validation for long-term OS (**A**, **C**) and CSS (**B**, **D**)

**Fig. 5:**
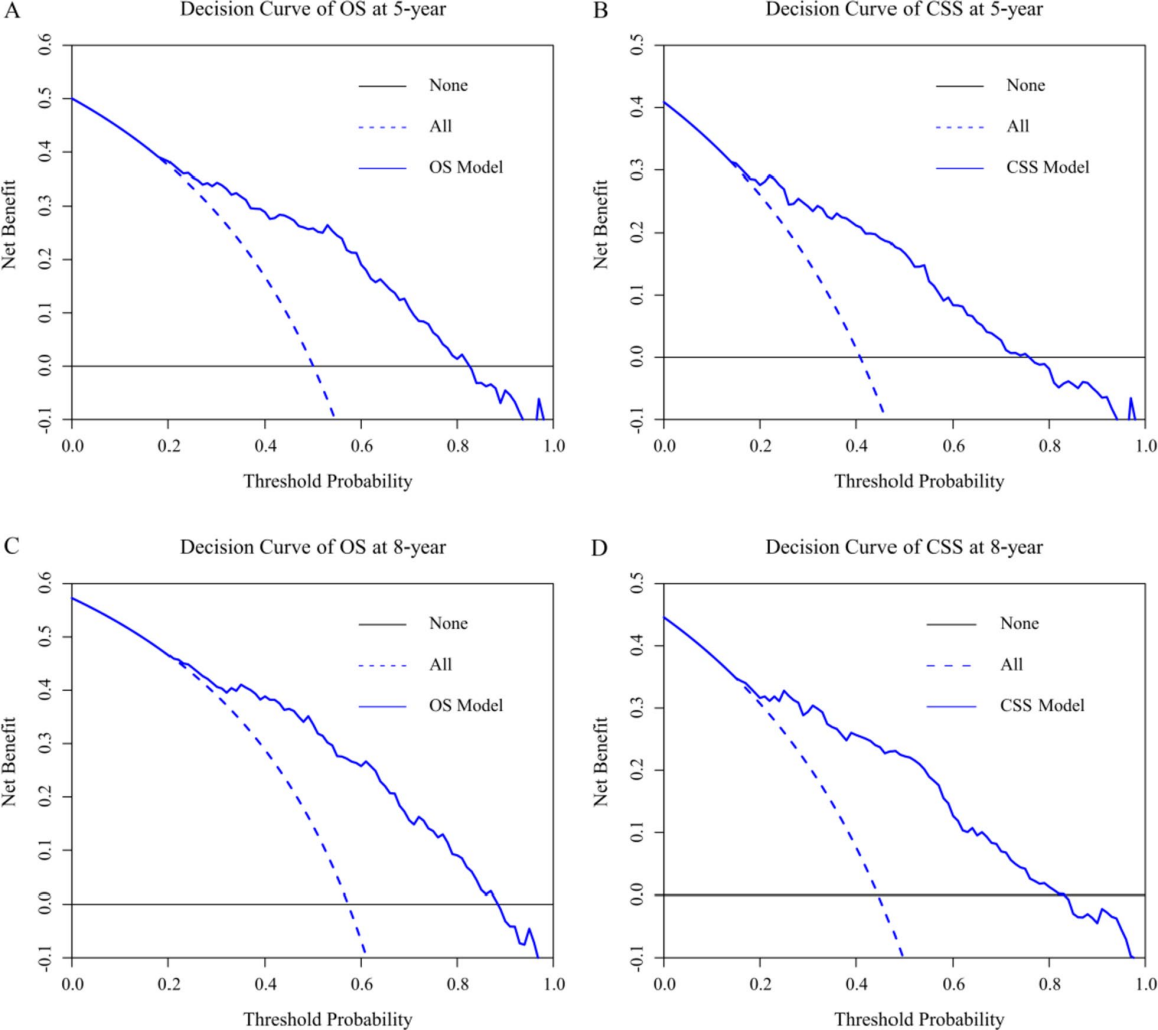
Decision curves of OS and CSS in validation group

The C-indexes of OS and CSS in internal validation were 0.782 (95% CI: 0.756–0.808) and 0.793 (95% CI: 0.763–0.823) respectively. External validation showed that the C-indexes of OS and CSS were 0.784 (95% CI: 0.754–0.814) and 0.785 (95% CI: 0.751–0.817), respectively. All the C-indexes were higher than 0.7, indicating excellent consistency. What’s more, OS and CSS nomograms’ calibration curves revealed that the observed 5- and 8-year survival probabilities were approach to the 45-degree reference line (Figs. 3, 4). Notably, the 5- and 8-year DCA curves showed that both nomogram models exerted net benefit in the validation group, demonstrating positive clinical value (Fig. 5).

## Discussion

Smoking, alcohol consumption, chewing betel quid increase the incidence of BMC around the world [10, 19]. Smoking has a deleterious impact on the development of oral cancer [20]. Cytomorphometry research shows that smoking has adverse effects on the buccal mucosa [21]. In a 10-year follow-up study of 12,212 people, smoking cessation reduced the incidence of oral mucosal lesions [22]. Epidemiological data shows that about 600 million people chew betel quid worldwide. The betel leaf, areca nut, and lime inside are important causes of the high incidence of BMC in the Pacific Islands, South Asia, and Southeast Asia [23, 24]. In India, risk habits such as chewing tobacco and putting the tobacco-containing quid into the gingivobuccal sulcus existed and BMC incidence accounted for 41% of oral cancers [25].

Usually, surgery, radiation and chemotherapy are the main modalities to treat BMC [26, 27]. Relevant literature shows that BMC is aggressive and the patients’ prognosis is poor [6]. Bachar et al. [28] has found that the overall recurrence rate was 41%, with 57.5% local control ratio. Hence, exploring risk factors related to the prognosis of patients with BMC has become increasingly important. Although many researchers have tried to reveal the prognostic factors, the main obstacle is the small sample size. In addition, BMC is always studied together with other oral cancers rather than conducted separately, leading to biased results [10]. Notably, the AJCC TNM staging manual is considered to be the most commonly used for prognostic assessment. However, many relevant parameters could affect the prognosis to a great extent. Therefore, personalized evaluation of BMC patients’ prognosis has emerged as an important trend. Numerous studies have proved that nomogram is fully qualified for patient-specific prognosis prediction [12]. To construct and validate the nomogram model, we applied the random split-sample method, which was used commonly and popularly [29, 30].

After the survival analysis, we found that age was of great importance to influence the OS and CSS, which is in agreement with the current research [31–33]. In our research, the age group “< 35 years” has the best OS and CSS, locating the far left of the age axis (Fig. 1). Tumor differentiation has a significant impact on the OS and CSS, which is in accordance with the research [34, 35]. TNM stages also play an important role in the prognosis of BMC patients [36, 37].

C-index is applied to measure the discrimination of the nomograms, with a scale from 0.5 to 1. In the process of validating the nomogram models, all the C-indexes are greater than 0.7, demonstrating high accuracy. Calibration curves are used to evaluate the fitting degree between the predicted probability of 5- and 8-year survival and observed risk [12]. In our research, the calibration curves fitted excellently with the diagonal reference line (Figs. 3, 4). Moreover, two nomogram’s decision curves possess good 5- and 8- year net benefit in the validation group, showing promising clinical value [38] (Fig. 5).

It is simple and practical to evaluate the individualized prognosis through nomogram. For example, nomogram graph includes various sub-categories axes. Each axis is marked with a different number. We draw a vertical line to the top point axis based on the personalized situation. In the same way, we add the points represented by each indicator to get the total points. Furthermore, we draw a vertical line from the total points axis to the 5- and 8- year OS and CSS axis to obtain the estimated survival rate. Notably, combined personalized total nomogram points with OS and CSS information, we acquire the optimal cut-off to divide patients into high- and low-risk cohorts (Fig. 2). Stateworthy, the nomogram was relatively more accurate than TNM staging. For instance, we set two T3N1M0 patients as an example (Additional files 1 and 2: Figs. 1 and 2). Patient 1: 60 years old, white, Grade II, Surgery, T3N1M0; Patient 2: 55 years old, black, Grade III, non-surgery, radiation, T3N1M0. If we evaluated the above patient’s prognosis using TNM staging, their prognosis is the same as each other. But the OS and CSS were different via our nomogram model. The 5-year OS of the two patients were 43% and 10%. 8-year OS of the two patients were 24% and lower than 10% respectively. Moreover, the 5-year CSS of the two patients was 48% and 10%. The 8-year CSS was 38% and lower than 10% respectively. Hence, personal prognosis evaluation is of great importance than merely the TNM staging manual.

Our research owes apparent advantages and limited drawbacks. We collected the detailed information of BMC patients from the credible SEER database. Based on the data, we conducted a survival analysis to obtain the independent prognostic risk factors and establish two nomograms. However, other relevant indicators such as comorbidity [39], alcohol [40], extracapsular spread [32], chemotherapy [41] are also important factors affecting the prognosis, which are not included in the SEER database. In addition, the SEER program hasn’t incorporated disease-free survival, cumulative survival and progression-free survival. Moreover, we haven’t applied the 8th AJCC Staging manual (published October 2017) as our cases spanned the period 2004–2013. Some patients’ T and N stages will be upstaged accordingly based on new protocols. As the lack of depth of invasion (DOI) and perineural invasion (PNI) information in SEER, it is impossible to restage each patient. Nevertheless, research shows that the c-indexes of the nomograms constructed using the 7th and 8th AJCC staging manuals are about the same [42]. More research is required to compare and validate the predictive power of nomogram models established applying 8th AJCC staging manual versions.

Traditionally, the prognostic estimate is based on the patients’ population. That makes individualized management a challenge for clinicians. The TNM staging system is widely accepted for prognostic estimation, although it only accounts for tumor factors, many other factors such as grade, age, sex, ethnicity are not considered though these are also very important. Clinicians need to incorporate all this information to estimate specific individual outcome empirically. Nomogram is a useful tool to incorporate all these factors in a quantitative manner. By summing up the scores of each risk factor, the 5- and 8-year OS and CSS of specific BMC patient can be predicted, which would enhance the screening and early intervention of controllable risk factors. In addition, the nomogram score is used to stratify patients by risk, so clinicians should lay more emphasis on high-risk patients with high malignancy and multiple risk factors, then plan individualized treatment modality and follow-up strategy.

This is a retrospective study and is limited by the fact that many risk factors are not included in the SEER database. In the future, multi-center prospective studies are to be conducted to validate and improve the nomogram of BMC for better individual treatment planning and prognosis assessment.

## Conclusions

In our study, we established two nomogram models successfully and the models demonstrated excellent discrimination, performance after verification.

## Supplementary Information

**Additional file 1.** OS and CSS predictions for the first patient mentioned in the discussion.

**Additional file 2.** OS and CSS predictions for the second patient mentioned in the discussion.

## Data Availability

Public access to the SEER database is open (https://seer.cancer.gov/data/). After completing the registration form and getting the SEER*Stat username, the SEER incidence data can obtain. Under reasonable circumstances, the data and analysis involved in this article can be acquired with the corresponding author.

## Abbreviations

BMC: Buccal mucosa cancer
SEER: Surveillance, epidemiology and end results
OS: Overall survival
CSS: Cancer-specific survival
C-index: Concordance index
DCA: The decision curve analyses
OSCC: Oral squamous cell carcinoma
NCCN: National comprehensive cancer network
DOI: Depth of invasion
PNI: Perineural invasion
