# Supplementary figures and images for: Nomogram predicting long-term overall and cancer-specific survival of patients with buccal mucosa cancer
Source: BMC Oral Health. 2022 Apr 22;22:138. doi: 10.1186/s12903-022-02147-9 (PMC9026892; doi:10.1186/s12903-022-02147-9)

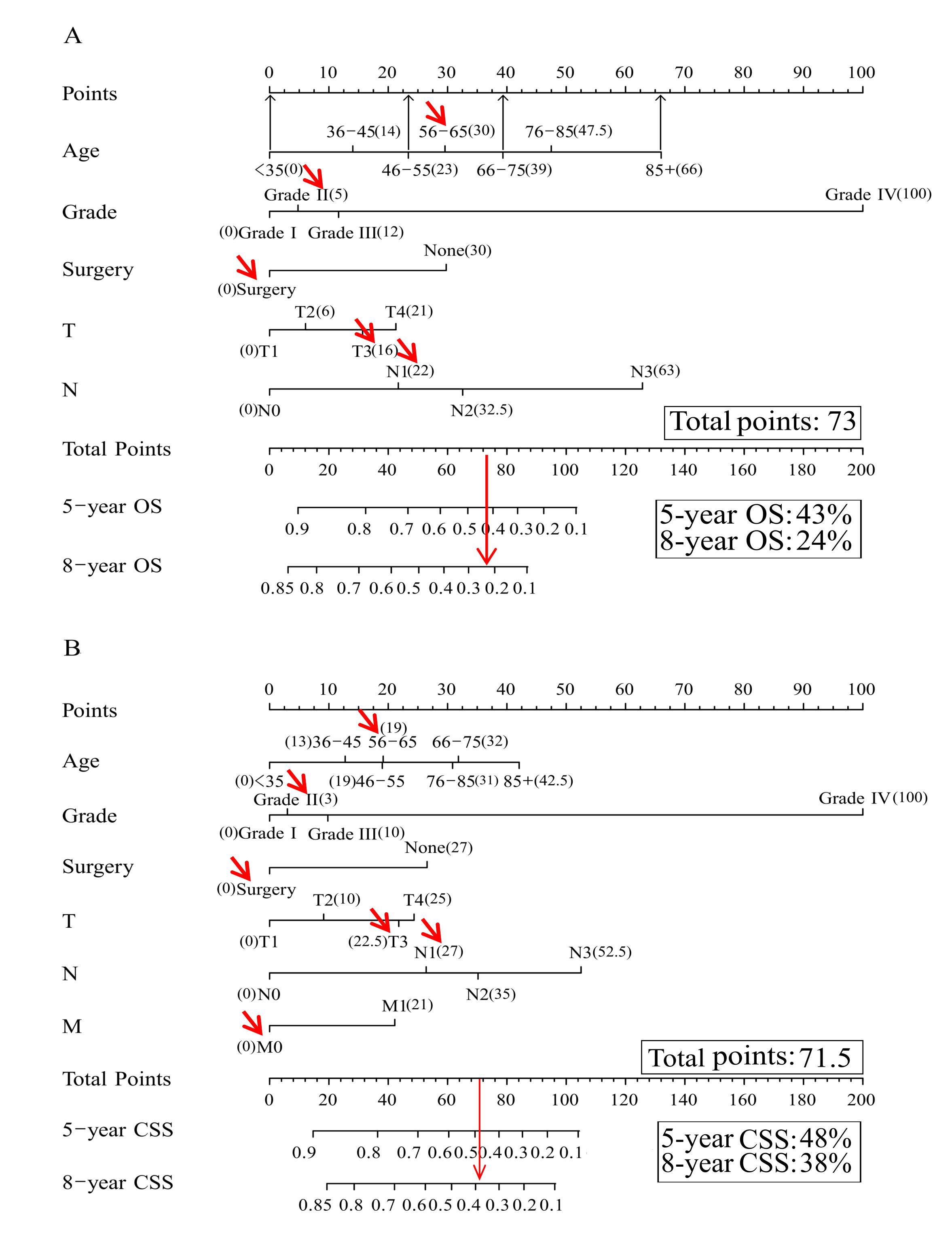

Supplement: Supplementary file 1 — Additional file 1. OS and CSS predictions for the first patient mentioned in the discussion. [file 12903_2022_2147_MOESM1_ESM.png]

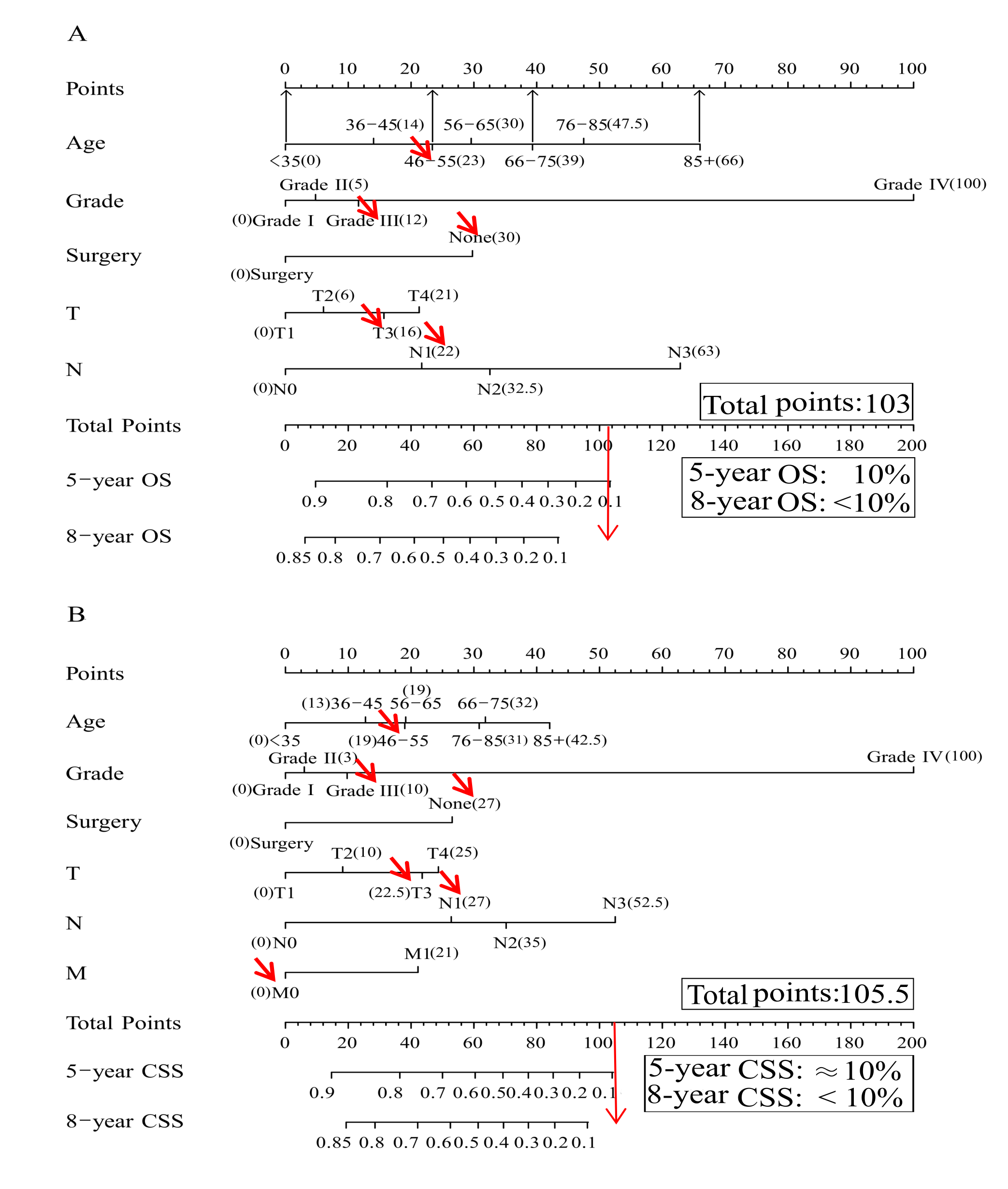

Supplement: Supplementary file 2 — Additional file 2. OS and CSS predictions for the second patient mentioned in the discussion. [file 12903_2022_2147_MOESM2_ESM.png]
